# Altered Circulating Immune Cell Distribution in Traumatic Spinal Cord Injury Patients in Relation to Clinical Parameters

**DOI:** 10.3389/fimmu.2022.873315

**Published:** 2022-06-28

**Authors:** Judith Fraussen, Lien Beckers, Charlotte C. M. van Laake-Geelen, Bart Depreitere, Jens Deckers, Erwin M. J. Cornips, Dieter Peuskens, Veerle Somers

**Affiliations:** ^1^ Department of Immunology and Infection, Biomedical Research Institute, Hasselt University, Hasselt, Belgium; ^2^ Adelante Centre of Expertise in Rehabilitation and Audiology, Hoensbroek, Netherlands; ^3^ Department of Rehabilitation Medicine, Research School CAPHRI, Maastricht University, Maastricht, Netherlands; ^4^ Division of Neurosurgery, University Hospitals Leuven, Leuven, Belgium; ^5^ Department of Neurosurgery, Algemeen Ziekenhuis (AZ) Turnhout, Turnhout, Belgium; ^6^ Department of Neurosurgery, Ziekenhuis Oost-Limburg, Genk, Belgium

**Keywords:** traumatic spinal cord injury, high-dimensional flow cytometry, B cells, CD74, immune profiling

## Abstract

Following a spinal cord injury (SCI), an inflammatory immune reaction is triggered which results in advanced secondary tissue damage. The systemic post-SCI immune response is poorly understood. This study aimed to extensively analyse the circulating immune cell composition in traumatic SCI patients in relation to clinical parameters. High-dimensional flow cytometry was performed on peripheral blood mononuclear cells of 18 traumatic SCI patients and 18 healthy controls to determine immune cell subsets. SCI blood samples were collected at multiple time points in the (sub)acute (0 days to 3 weeks post-SCI, (s)aSCI) and chronic (6 to >18 weeks post-SCI, cSCI) disease phase. Total and CD4^+^ T cell frequencies were increased in cSCI patients. Both CD4^+^ T cells and B cells were shifted towards memory phenotypes in (s)aSCI patients and cSCI patients, respectively. Most profound changes were observed in the B cell compartment. Decreased immunoglobulin (Ig)G^+^ and increased IgM^+^ B cell frequencies reflected disease severity, as these correlated with American Spinal Injury Association (ASIA) impairment scale (AIS) scores. Post-SCI B cell responses consisted of an increased frequency of CD74^+^ cells and CD74 expression level within total B cells and B cell subsets. Findings from this study suggest that post-SCI inflammation is driven by memory immune cell subsets. The increased CD74 expression on post-SCI B cells could suggest the involvement of CD74-related pathways in neuroinflammation following SCI. In addition, the clinical and prognostic value of monitoring circulating IgM^+^ and IgG^+^ B cell levels in SCI patients should be further evaluated.

## Introduction

Spinal cord injury (SCI) causes irreversible damage to the nerve tissue of the spinal cord, which results in the loss of motor and sensory functions at and below the injury level (1). An inflammatory and autoreactive immune reaction is triggered following a SCI, leading to both protective effects and further tissue damage during a secondary injury phase (2–8).

The innate immune system provides an immediate response during inflammation. As seen in post-mortem human SCI tissue, neutrophils and microglia are the first immune cells to enter the lesion site (9). Monocytes start to infiltrate the spinal cord 1-3 days post-SCI (9). Foamy macrophages, derived from both resident microglia and monocytes, remain present in the injured spinal cord up to one year post-SCI (6, 9). The adaptive immune response unfolds later since it takes more time for lymphocytes to recognize their specific antigen, become activated, proliferate and undergo clonal expansion. As a consequence, CD4^+^ helper and CD8^+^ cytotoxic T cells are sparse in the spinal cord during the acute SCI stage and increase in numbers in chronic SCI (>1 month post-SCI) (9). Although B cells could only be detected in the spinal cord of a subset of SCI patients (10), autoreactive antibodies directed against spinal cord proteins, glycoproteins and lipids have been identified in the serum and cerebrospinal fluid (CSF) of SCI patients (11–18). In addition, evidence from experimental models indicates a detrimental role for B cells in SCI that can, at least partly, be explained by the production of pathogenic antibodies (8, 19, 20).

Local activation and differentiation of adaptive immune cells inside the injured spinal cord was indicated by the finding of ectopic lymphoid follicles in the spinal cord of SCI mice (21). Peripheral immune activation was shown in experimental SCI as well, as numbers of cytokine-producing T cells were elevated in the spleen post-SCI (2). In humans, it is largely unknown how SCI affects the peripheral immune composition. More detailed knowledge on changes in immune cell populations, the timing of these changes following the injury and possible associations with clinical characteristics would aid in the clinical evaluation and therapeutic management of SCI. The aim of the current study was to extensively analyse the circulating immune cell composition for the first time during both the (sub)acute and chronic stages of post-SCI inflammation by high-dimensional flow cytometry and its correlation with clinical parameters. In addition, B cell survival receptor expression was studied in order to identify potentially relevant pathways for the production of autoantibodies that were shown to contribute to post-SCI tissue degeneration.

## Materials and Methods

### Study Subjects

SCI patients were recruited at the Ziekenhuis Oost-Limburg (Genk, Belgium), University Hospitals Leuven (Leuven, Belgium), AZ Turnhout (Turnhout, Belgium), Adelante Centre of Expertise in Rehabilitation (Hoensbroek, The Netherlands) and Maastricht UMC+ (Maastricht, The Netherlands). Healthy controls (HC) were recruited at Hasselt University (Hasselt, Belgium). The study was approved by the institutional Medical Ethics Committees. Written informed consent was obtained from all participants. Samples were cryopreserved at the University Biobank Limburg.

Peripheral blood was collected from traumatic SCI patients, who were not treated with corticosteroids, and HC (n=18 each, **Table 1**). In total, 46 SCI samples were included at different time points: t0 (0-4 days post-SCI, n=9), t1 (3 weeks post-SCI, n=8), t2 (6 weeks post-SCI, n=9), t3 (12 weeks post-SCI, n=10), t4 (18 weeks post-SCI, n=7) and t5 (>18 weeks post-SCI, n=3). A detailed overview of the individual SCI patients is given in **Supplementary Table 1**. The first 2 time points (t0-t1) were considered to represent the (sub)acute disease phase ((s)aSCI, ≤1 month post-SCI), while the others (t2-t3-t4-t5) were considered to be chronic time points (cSCI, >1 month post-SCI). Injury severity, as measured by the American Spinal Injury Association (ASIA) impairment scale (AIS), was balanced in the (s)aSCI and cSCI groups. The AIS is a standardized examination consisting of a myotomal-based motor examination, dermatomal-based sensory examination, and an anorectal examination aimed at determining SCI severity, in which score A represents a complete injury, B a sensory incomplete injury, C a motor incomplete injury with less than half of key muscles below the neurological level having a muscle grade greater than or equal to 3, D a motor incomplete injury with at least half of key muscles below the neurological level having a muscle grade greater than or equal to 3 and E represents normal functioning (22). HC did not have allergies, autoimmune disorders or infections at the moment of sampling and were matched to SCI patients with regard to age and sex as closely as possible.

**Table 1:** Study cohort.

	HC	SCI
**n**	18	18
**Gender (n males, %)**	16 (89)	16 (89)
**Age (years, SD)**	56 (13)	56 (15)
**Level of injury (n, %)**		
** Cervical**	n.a.	12 (67)
** Thoracic**	n.a.	5 (28)
** Lumbar**	n.a.	1 (5.5)
**Injury mechanism (n, %)**		
** Motor vehicle accident**	n.a.	3 (17)
** Fall**	n.a.	11 (61)
** Sports accident**	n.a.	3 (17)
** Gunshot**	n.a.	1 (5)
**AIS score at t0 (n, %)**		
** A**	n.a.	6 (33)
** B**	n.a.	3 (17)
** C**	n.a.	3 (17)
** D**	n.a.	6 (33)
**Motor score^a,b^ at first sampling**	n.a.	50 (10-95)
**Pinprick score^a,c^ at first sampling**	n.a.	80 (18-109)
**Light touch score^a,d^ at first sampling**	n.a.	80 (36-109)
**Surgery (n, %)**	n.a.	17 (94)
**Included timepoints (n, %)**		
** t0**	n.a	9 (50)
** t1**	n.a	8 (44)
** t2**	n.a	9 (50)
** t3**	n.a	10 (56)
** t4**	n.a	7 (39)
** t5**	n.a	3 (17)

a median (range).

b available for 11/18 patients.

c available for 7/18 patients.

d available for 8/18 patients.

### Cell Isolation

Peripheral blood mononuclear cells (PBMC) were isolated from whole blood by Ficoll density gradient centrifugation (Lympholyte; Cedarlane Laboratories, SanBio B.V., Uden, The Netherlands). Cryopreserved PBMC were used for flow cytometry.

### Flow Cytometry

Cryopreserved PBMC were thawed, immediately recovered in 20% fetal bovine serum (FBS, Gibco, Thermo Fisher Scientific, Waltham, MA, USA) in RPMI 1640 (Lonza, Basel, Switzerland) with 0.5mg/ml DNAse (Merck Life Science BV, Hoeilaart, Belgium) and counted. General immune cell subsets and T cell subsets were measured using 0.5 x 10^6^ PBMC as previously described (23). B cell subsets and their expression of survival markers were analyzed using 1 x 10^6^ PBMC. First, PBMC were washed two times in phosphate-buffered saline (PBS) and stained with the fixable viability dye (FVD) eFluor506 (eBioscience, Thermo Fisher Scientific, Bleiswijk, The Netherlands) during 30 min. Next, surface markers were stained during 15 min using anti-human monoclonal antibodies (**Supplementary Table 2**) against CD19, CD24, CD38, CD27, CD74, IgD, IgM, CD267 (TACI, transmembrane activator calcium modulator and cyclophilin ligand interactor), CD268 (B cell activating factor receptor, BAFF-R), CD269 (B cell maturation antigen, BCMA) (all from BioLegend, Amsterdam, The Netherlands), IgG (BD Biosciences, Erembodegem, Belgium) and IgA (Miltenyi Biotec, Leiden, The Netherlands). Fluorescence minus one (FMO) controls were used for gating. Flow cytometry was performed on a LSRFortessa flow cytometer (BD Biosciences). SCI samples collected at different time points, as well as matched HC and SCI samples, were simultaneously analyzed to exclude inter-assay variation. The number of samples included in each group ((s)aSCI, cSCI) differed between flow cytometric analyses due to limitations in cell numbers for some samples (priority for B cell subset analysis, followed by general immune cell subsets and T cell subsets) and is specified in the figure legends and in the first graph of each figure panel.

### Macrophage Migration Inhibitory Factor ELISA

Macrophage migration inhibitory factor (MIF) levels in the plasma were measured using the Human MIF ELISA Kit (Fisher Scientific, Aalst, Belgium), according to the instructions of the manufacturer. Plasma (50µl) was diluted twofold with Assay Diluent before analysis. All samples were analyzed in duplicate.

### Multidimensional Flow Cytometry Data Analysis

Flow cytometry data was analyzed using FlowJo v10.6.2 (FlowJo, LLC, BD Life Sciences, Oregon, USA). First, single cells or lymphocytes from each individual were randomly down-sampled to an equal number of events (10,000) for general immune cell subsets or T cell subsets, respectively. All events were included in the analysis of B cell subsets. Individual files were then concatenated into a single file that still allowed the discrimination of the study groups. Dimensionality reduction was performed using the t-Distributed Stochastic Neighbor Embedding (tSNE) native platform in FlowJo with 1,000 iterations. Clustering of the data using self-organizing maps (SOM) was done using FlowSOM v2.6 with grid size of 10x10 (24). The number of metaclusters was 10 for general immune cell subsets, 15 for T cell subsets and 12 for B cell subsets.

### Statistical Analysis

Data analysis was done using Prism software version 8.4 (GraphPad Software, San Diego, CA, USA) and JMP Pro 14.2 software (SAS Institute, Cary, NC, USA). Differences in immune cell subsets and MIF levels between the study groups were analyzed using a linear mixed model, allowing for repeated measurements and missing values. Age, injury severity (AIS) and injury level (cervical, thoracic) were included as covariates. Patient ID was included as a random effect to account for the association of measurements within the same patient. When necessary, continuous variables were log-transformed to achieve a Gaussian distribution. Acute and subacute SCI patients were first analyzed for differences in immune cells and MIF levels. As differences were only found for IgA^+^ B cells, TACI^+^ class-switched memory (CSM) and TACI^+^ double negative (DN) B cells, acute and subacute SCI patients were combined into a (s)aSCI group for analysis of the other parameters. Correlations between immune cell frequencies and AIS scores at the sampling timepoint were done using Spearman correlation tests. Differences in the distribution of AIS scores between (s)aSCI and cSCI groups were tested using Fisher’s Exact test. A p-value of <0.05 was considered significant.

## Results

### General Immune Profiling Indicates Increased CD4^+^ T Cells During cSCI

**Figure 1:**
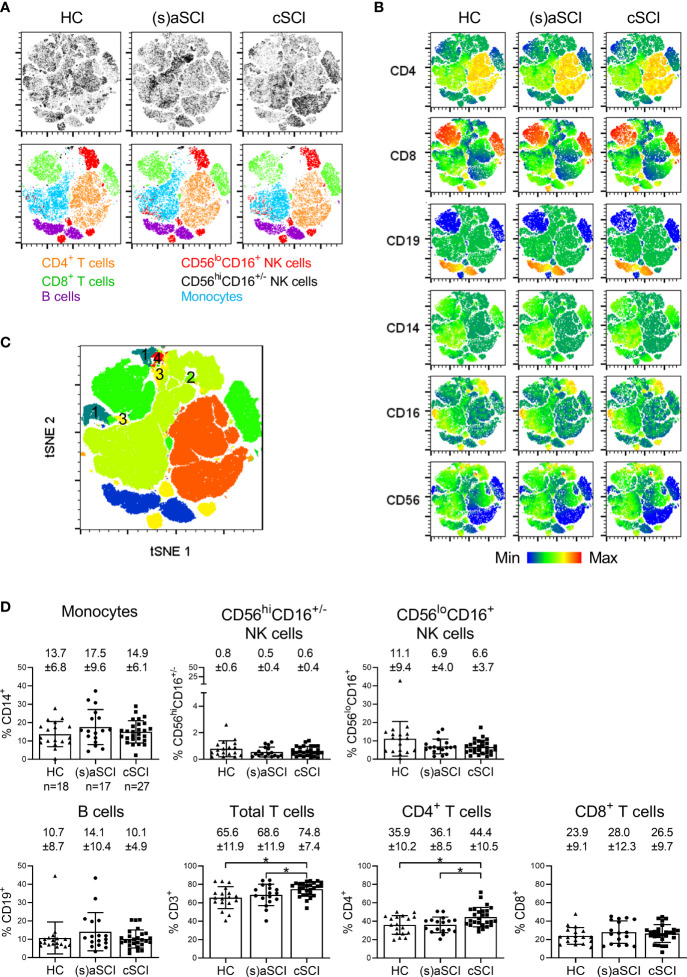
Frequency of general immune cell subsets in the peripheral blood of HC and SCI patients. **(A)** tSNE maps showing the major immune cell subsets within single cells of HC (n = 18) and SCI patients (n = 18), including (s)aSCI (17 samples) and cSCI (27 samples). In the upper row, tSNE maps are shown on a density plot while the lower row shows the manually determined gates on the tSNE maps. **(B)** Surface marker distribution is depicted in the tSNE map. Relative antigen expression is visualized by the color tone (from blue to red). **(C)** FlowSOM clusters are shown in the tSNE map as overlay. The clusters that were not included in our manual gating strategy are numbered from 1 to 4. **(D)** Percentages of monocytes, CD56^hi^CD16^+/-^ NK cells, CD56^lo^CD16^+^ NK cells, B cells, total CD3^+^ T cells, CD4^+^ T cells and CD8^+^ T cells are shown within the lymphocyte population. Mean (bars) ± SD is depicted and shown in numbers. The number of included samples in each group is shown in the first graph. *p < 0.05.

We first analyzed the major immune cell subsets in PBMC of HC, (s)aSCI patients and cSCI patients. These included CD14^+^ monocytes, natural killer (NK) cells, which are classically divided into cytotoxic CD56^lo^CD16^+^ and highly cytokine producing CD56^hi^CD16^+/-^ subsets, CD19^+^ B cells and CD3^+^ T cells, divided into CD4^+^ helper and CD8^+^ cytotoxic subsets. tSNE and FlowSOM algorithms were used in an exploratory manner to visualize the different immune cell subsets. Density tSNE plots of the major immune cell subsets showed differences in density and thus in immune cell distribution between the study groups (**Figure 1A** upper row). An overlay of the manually gated immune cells on the tSNE maps showed separate clustering of the major immune cell subsets (**Figure 1A** lower row). Moreover, the differences in immune cell distribution could be retraced to all included subsets (**Figures 1A** **, B**). FlowSOM analysis provided an additional control and corresponded well to the tSNE map (**Figure 1C**), as metaclustered cells were uniformly arranged into the distinct tSNE areas with a strong agreement of marker expression. As indicated by numbering, 4 additional immune cell subsets were identified in FlowSOM analysis, including CD4^-^CD8^-^ T cells (1) and CD16^+/-^CD56^+^ NK T cells (2–4). Comparison of the study groups showed a significant increase in the frequencies of total CD3^+^ T cells and CD4^+^ T cells in cSCI patients when compared with both (s)aSCI patients (p=0.0478, 95% CI [0.0006,0.129] and p=0.0037, 95% CI [0.025,0.116], respectively) and HC (p=0.0126, 95% CI [0.019,0.149] and p=0.017, 95% CI [0.017,0.164], respectively) (**Figure 1D**). No significant differences were found in the frequencies of monocytes, NK cells, B cells or CD8^+^ T cells between HC, (s)aSCI and cSCI patients. Thus, total and CD4^+^ T cells were increased during the chronic phase of a SCI.

### The Peripheral Blood Distribution of CD4^+^ T Cell Subsets Is Altered Following a SCI

**Figure 2:**
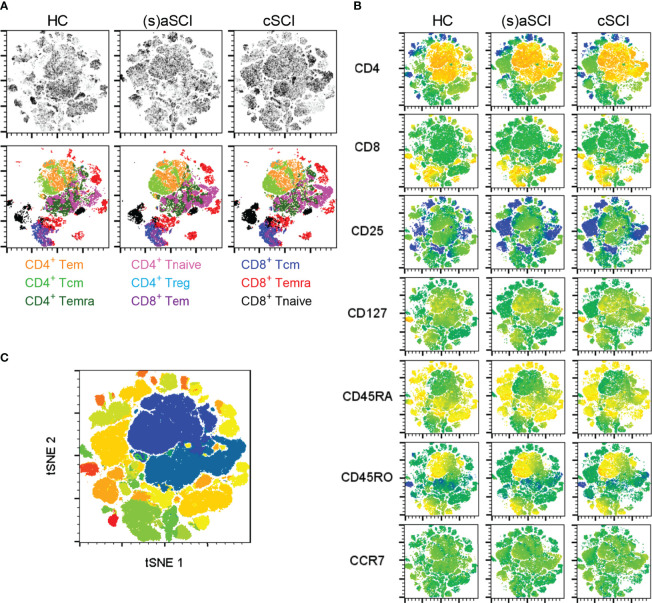
High-dimensional flow cytometry of T cell subsets in the peripheral blood of HC and SCI patients. **(A)** tSNE maps showing T cell subsets within the lymphocyte population of HC (n = 18) and SCI patients (n = 18), including (s)aSCI (16 samples) and cSCI (28 samples). In the upper row, tSNE maps are shown on a density plot while the lower row shows the manually determined gates on the tSNE maps. **(B)** Surface marker distribution is depicted in the tSNE map. Relative antigen expression is visualized by the color tone (from blue to red). **(C)** FlowSOM clusters are shown in the tSNE map as overlay. FlowSOM analysis was performed on the lymphocyte population as no general T cell marker was included in the flow cytometry panel.

**Figure 3:**
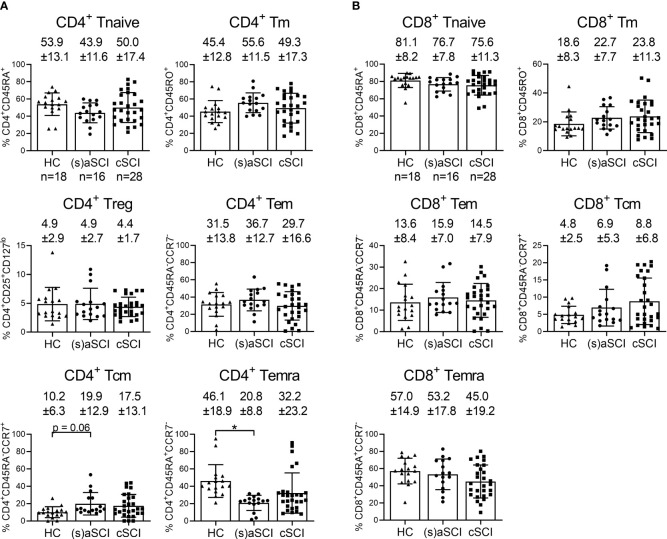
Frequency of T cell subsets in the peripheral blood of HC and SCI patients. Percentages of CD4^+^ **(A)** and CD8^+^ **(B)** Tnaive, Tm, Tem, Tcm and Temra are shown within the lymphocyte population of HC (n = 18) and SCI patients (n = 18), including (s)aSCI (16 samples) and cSCI (28 samples), as indicated in the first graph of each figure panel. Mean (bars) ± SD is depicted and shown in numbers. *p < 0.05.

As differentially expressed immune cell subsets could be retraced to all major immune cell populations (**Figures 1A** **, B**), more detailed immune profiling was warranted. The following subsets were studied in both CD4^+^ and CD8^+^ T cells: naive (Tnaive, CD45RA^+^CD45RO^-^), memory (Tm, CD45RA^-^CD45RO^+^), effector memory (Tem, CD45RA^-^CCR7^-^), central memory (Tcm, CD45RA^-^CCR7^+^), effector memory re-expressing CD45RA (Temra, CD45RA^+^CCR7^-^) and regulatory (Treg, CD25^+^CD127^low^). tSNE analysis showed separate clustering of CD4^+^ and CD8^+^ T cells and in each of these subsets also of memory (CD45RO^+^) and naive (CD45RA^+^) phenotypes (**Figure 2A** **, B**). Metaclusters generated by FlowSOM corresponded well with tSNE analysis (**Figure 2C**). Most CD8^+^ T cells were Tnaive in HC (81.1 ± 8.2%), (s)aSCI (76.7 ± 7.8%) and cSCI (75.6 ± 11.3%) patients (**Figure 3A** **, B**). For CD4^+^ T cells, however, the distribution shifted from more Tnaive in HC (53.9 ± 13.1% Tnaive vs 45.4 ± 12.8% Tm) to more Tm in (s)aSCI patients (43.9 ± 11.6% Tnaive vs 55.6 ± 11.5% Tm). Further, HC showed a significantly higher proportion of CD4^+^ Temra (p=0.032, 95% CI [0.015,0.379]) and a trend towards a decreased frequency of CD4^+^ Tcm (p=0.061, 95% CI [-0.198,0.004]) compared with (s)aSCI patients. For CD4^+^ T cell subsets, a weak correlation was only observed between the frequency of CCR7^+^ naive T cells (p=0.0277, r=0.3319) and AIS disease score (**Supplementary Figure 1**). Together, these results showed similar CD4^+^ and CD8^+^ T cell subset frequencies in HC and SCI patients, except for a trend towards more CD4^+^ Tm and less CD4^+^ Temra in (s)aSCI patients versus HC.

### The B Cell Compartment of cSCI Patients Reflects an Increase in Memory Responses

**Figure 4:**
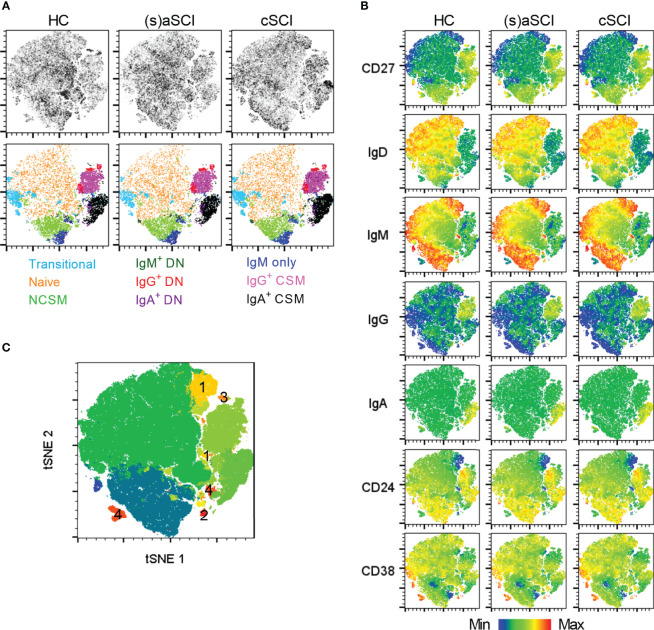
High-dimensional flow cytometry of B cell subsets in the peripheral blood of HC and SCI patients. **(A)** tSNE map showing B cell subsets within the CD19^+^ B cell population of HC (n = 18) and SCI patients (n = 18), including (s)aSCI (17 samples) and cSCI (29 samples). In the upper row, tSNE maps are shown on a density plot while the lower row shows the manually determined gates on the tSNE maps. **(B)** Surface marker distribution is depicted in the tSNE map. Relative antigen expression is visualized by the color tone (from blue to red). **(C)** FlowSOM clusters are shown in the tSNE map as overlay. The clusters that were not included in our manual gating strategy are labelled from 1 to 4.

**Figure 5:**
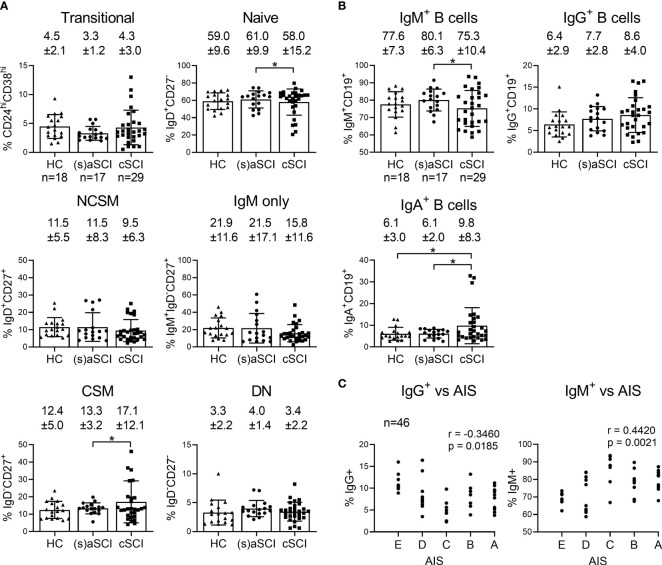
Frequency of B cell subsets in the peripheral blood of HC and SCI patients. Percentages of the major B cell subsets **(A)** and IgM/G/A expressing B cells **(B)** are shown within the lymphocyte population of HC (n = 18) and SCI patients (n = 18), including (s)aSCI (17 samples) and cSCI (29 samples). Mean (bars) ± SD is depicted and shown in numbers. **(C)** Correlation of the IgG^+^ or IgM^+^ B cell frequency and AIS score at sampling timepoint. r denotes the Spearman correlation coefficient (rho). Numbers of included samples in each group are also indicated in the first graph of each figure panel. *p < 0.05.

Next, CD19^+^ B cells were studied in more detail by analysis of CD24^hi^CD38^hi^ transitional, IgD^+^CD27^-^ naive, IgD^+^CD27^+^ non class-switched memory (NCSM), IgD^-^CD27^+^ class-switched memory (CSM), IgM^+^IgD^-^CD27^+^ IgM only and IgD^-^CD27^-^ double negative (DN) subsets. IgM, IgG and IgA were included to further define memory responses. Interestingly, tSNE maps showed that B cell subsets clustered together based on Ig expression and not on the expression of subset-defining surface markers such as CD27 and IgD (**Figures 4A** **, B**). In this regard, IgM^+^ memory subsets (NCSM, DN, IgM only) formed separate clusters compared with IgG^+^ (DN, CSM) and IgA^+^ (DN, CSM) memory subsets. Furthermore, a highly dense cluster of IgA^+^ CSM B cells was present in cSCI patients when compared with HC and (s)aSCI patients (**Figure 4A** upper row). In FlowSOM analysis, which corresponded well to tSNE analysis (**Figure 4C**), multiple metaclusters were representative of B cell subsets that were not included in the manual gating analysis (numbered 1-4). These subsets had naive (1) or IgM only memory (2) phenotypes or were undefined B cell subsets (3–4) characterized by CD27 and CD38 expression and the absence of Ig expression. A decreased frequency of naive B cells (p=0.027, 95% CI [-0.078,-0.005]) and an increased frequency of CSM B cells (p=0.032, 95% CI [0.002,0.049]) was indicated in cSCI patients when compared with (s)aSCI patients, the latter showing similar frequencies of naive (p=0.61, 95% CI [-0.107,0.064]) and CSM (p=0.52, 95% CI [-0.079,0.041]) B cells as HC (**Figure 5A**). This shift to a memory B cell phenotype during chronic SCI was also shown in the decreased frequency of IgM^+^ B cells in cSCI patients compared to (s)aSCI patients (p=0.04, 95% CI [-0.103,-0.003]) and in the increased frequency of IgA^+^ B cells in cSCI patients compared to both (s)aSCI patients (p=0.012, 95% CI [0.069,0.528]) and HC (p=0.029, 95% CI [0.045,0.770]) (**Figure 5B**). Of note, the frequency of IgA^+^ B cells was significantly lower in acute versus subacute SCI patients (p=0.034, 95% CI [-0.561,-0.028], **Supplementary Figure 2**). Interestingly, a negative correlation was shown between the frequency of IgG^+^ B cells and injury severity (p=0.0185, r=-0.3460) and a positive correlation between IgM^+^ B cells and injury severity (p=0.0021, r=0.4420), as indicated by AIS score (**Figure 5C**). As expected, IgG^+^ B cells were inversely correlated with IgM^+^ B cells (p<0.0001, r=-0.7464, **Supplementary Figure 3A**). The frequency of IgM^+^ B cells was also significantly lower in SCI patients with a cervical injury (74.2 ± 9.2%) versus those with a thoracic injury (84.5 ± 5.7%, p=0.0415, 95% CI [-0.236,-0.005], **Supplementary Figure 3B**). Overall, these results indicate a shift towards more memory B cell subsets in the peripheral blood of cSCI patients. Moreover, the frequency of circulating IgG^+^ and IgM^+^ B cells could potentially be used as an indicator of disease severity.

### CD74 Might Contribute to Post-SCI B Cell Responses

**Figure 6:**
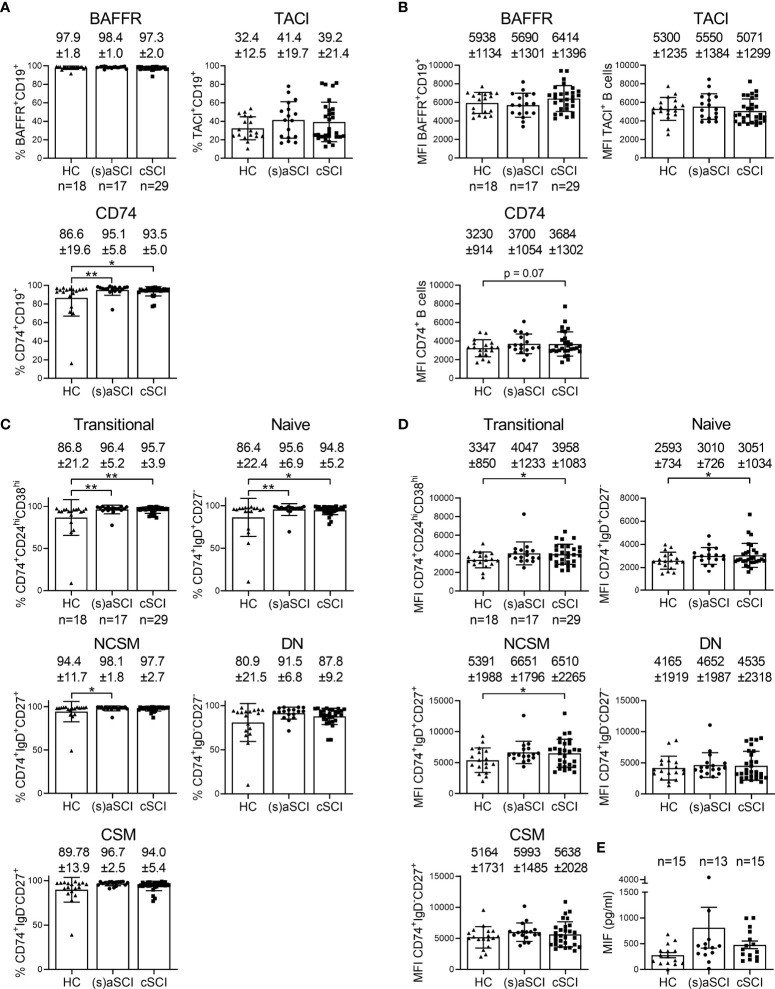
Involvement of B cell survival receptor expression in post-SCI immunity. Percentages **(A)** and median fluorescence intensity (MFI) **(B)** of B cells expressing BAFFR, TACI and CD74 are shown within the B cell population of HC (n = 18) and SCI patients (n = 18), including (s)aSCI (17 samples) and cSCI (29 samples). Mean (bars) ± SD is depicted and shown in numbers. Percentages **(C)** and MFI **(D)** of CD74 expression is shown for transitional, naive, NCSM, DN and CSM B cell subsets of HC (n = 18) and SCI patients (n = 18), including (s)aSCI (17 samples) and cSCI (29 samples). **(E)** The concentration of MIF (pg/ml) in the plasma is shown for HC (n = 15) and SCI patients (n = 18), including (s)aSCI (n = 13 samples) and cSCI (n = 15 samples). Mean (bars) ± SD is depicted. Numbers of included samples in each group are also indicated in the first graph of each figure panel. *p < 0.05, **p < 0.01.

In order to identify potentially relevant pathways for the production of autoantibodies, which have already been shown to contribute to post-SCI tissue degeneration, the expression of B cell survival receptors was evaluated. More specifically, expression of BAFFR (receptor for the survival factor BAFF), TACI (receptor for the survival factors BAFF and a proliferation-inducing ligand, APRIL) and CD74 (receptor for macrophage migration inhibitory factor, MIF) was measured on total B cells and B cell subsets. B cell maturation antigen (BCMA) was evaluated as well but its expression on circulating B cells was negligible (data not shown). Acute SCI patients showed a significantly decreased frequency of TACI^+^ CSM and DN B cells when compared with subacute SCI patients (p=0.019, 95% CI [-0.289,-0.038] and p=0.008, 95% CI [-0.164,-0.037], respectively, **Supplementary Figure 2**). The frequency of CD74^+^ B cells was significantly increased in the peripheral blood of (s)aSCI (p=0.0067, 95% CI [-0.167,-0.028]) and cSCI (p=0.0443, 95% CI [0.002,0.125]) patients compared to HC (**Figure 6A**). There was a trend towards increased CD74 expression (median fluorescence intensity, MFI) on circulating total B cells of cSCI patients compared with HC (p=0.07) (**Figure 6B**). Furthermore, the frequency of CD74^+^ cells was increased within all circulating B cell subsets of (s)aSCI and cSCI patients compared to HC and was statistically significant for transitional and naive B cells in (s)aSCI patients (p=0.0025, 95% CI [-0.183,-0.041] and p=0.0033, 95% CI [-0.195,-0.041], respectively) and cSCI patients (p=0.0075 and p=0.0180, 95% CI [0.015,0.154], respectively) and for NCSM B cells in (s)aSCI patients (p=0.0405, 95% CI [-0.107,-0.001]) (**Figure 6C**). In addition, CD74 expression was increased on transitional (p=0.0348, 95% CI [55.05,1427.36]), naive (p=0.0288, 95% CI [0.023,0.402]) and NCSM (p=0.0325, 95% CI [130.31,2858.68]) B cells of cSCI patients compared to HC (**Figure 6D**). As CD74 is the receptor for MIF on the B cell surface, we additionally evaluated plasma MIF levels in HC and SCI patients. Plasma MIF levels were not different between the study groups (**Figure 6E**). Together, these results suggest that CD74-related pathways might be involved in post-SCI B cell immune responses.

## Discussion

In this study, we performed a detailed immune profiling of SCI patients in the (sub)acute and chronic disease phase using high-dimensional flow cytometry. Changes in the frequencies of circulating immune cell subsets were identified in SCI patients compared to HC, reflecting more memory T and B cell responses in SCI patients. Most profound changes were present in the B cell compartment, where decreased IgG^+^ and increased IgM^+^ B cell frequencies correlated with injury severity. Post-SCI B cell responses had a memory phenotype and were characterized by increased CD74 expression.

General immune profiling indicated an increased frequency of total and CD4^+^ T cells in cSCI patients compared to HC and (s)aSCI patients. This is in agreement with a cross-sectional report of cSCI patients at varying, mostly chronic, time points that additionally showed a decrease in the circulating frequency and *in vitro* cytotoxicity of CD16^+^CD56^+^ NK cells (25). It also corresponds with the increase in CD4^+^ T cells in post-mortem SCI tissue weeks to months after initial injury (9). Another study described a rapid decrease in circulating monocytes, T cells and B cells for 1 week post-SCI and a return to control levels in the chronic phase (105-136 days post-SCI) (26). No additional proportional changes were demonstrated in the major immune cell subsets in our study. More detailed flow cytometry on T cell subsets indicated an elevation in circulating CD4^+^ Tm cells in (s)aSCI patients, decrease in CD4^+^ Temra cells and increase in CD4^+^ Tcm cells. Elevated proportions of memory CD4^+^ T cell subsets are also seen in autoimmune diseases such as multiple sclerosis and psoriasis where these T cells are repeatedly activated by autoantigens and therefore undergo expansion (27). Thus, our results indicate an ongoing activation and expansion of memory CD4^+^ T cells in SCI patients due to repeated (auto)immune activation and highlight the importance of this subset in mediating (sub)acute post-SCI systemic immune responses. CD4^+^ Temra denotes a subset of effector memory T cells which play an important role in T cell responses to viral pathogens (28). Thus, the observed T cell subset changes could possibly contribute to the immune depression that is observed in acute experimental SCI, although this depends on the injury level and severity (29). Functional and antigen-specific T cell subset analysis post-SCI is warranted to study this in more detail. As only a weak correlation was observed between the frequency of CCR7^+^ naive T cells and injury severity, inclusion of additional SCI patients is needed to confirm these results.

Similar to the T cell compartment, B cell phenotyping indicated a shift towards memory B cell responses in SCI patients, probably again reflecting repeated immune activation. This shift in B cell subsets was only evident in cSCI patients, indicating that similar proportional changes occur in the T and B cell compartments at different time points post-SCI. The later appearance of elevated memory B cells can be explained by the fact that their development takes longer than that of memory T cells. B cell activation in T cell dependent immune responses cannot begin until antigen-specific follicular helper CD4^+^ T cells are available, and B cells must then enter different phases of proliferation, selection and maturation in lymphoid tissue (germinal center response) (30–33). Interestingly, already with the current numbers of included SCI patients, IgG^+^ B cell levels were negatively correlated with injury severity while IgM^+^ B cells were positively correlated with injury severity. This could point towards the relocation of IgG^+^ B cells to the spinal cord, reflecting a higher proportion of IgG^+^ B cells in the spinal cord of SCI patients with more severe disease compared to those with less severe disease. CSF analyses of B cell subsets would be recommended for confirmation of IgG^+^ B cell relocation. However, ethical considerations complicate this analysis as lumbar punctures are not routinely performed post-SCI. For SCI management, monitoring circulating IgM^+^ and IgG^+^ B cell frequencies is feasible and could potentially be relevant for prognosis and clinical evaluation. We also evaluated correlations between immune cell frequencies and the individual AIS motor, pinprick and light touch scores, although no statistically significant correlations were found. This is probably due to the incomplete AIS subscore dataset for our cohort.

Another important finding was the increased frequency of CD74^+^ cells and CD74 expression level within total B cells and B cell subsets from SCI patients. CD74 expression was lowest on transitional and naive B cells, which corresponds to previous observations in multiple sclerosis (MS) (34). Together with CD44, cell surface CD74 is the receptor for MIF (35). CD74/CD44 activation by MIF induces cell entry into the S phase of the cell cycle, resulting in cell proliferation and increased expression of survival genes such as Bcl-X_L_ (36). Furthermore, MIF promotes B cell migration following engagement of CD74 and C-X-C chemokine receptor (CXCR)4 (37). Thus, our results suggest that CD74-related pathways might be involved in B cell migration and survival post-SCI, and thus possibly also in post-SCI autoantibody production. However, validation of our results in a larger cohort of SCI patients and controls is needed. Involvement of MIF in SCI pathology has been indicated in multiple studies. MIF expression was increased following contusion SCI in rats, where it elicited inflammatory astrocyte responses (38, 39). In a compression SCI mouse model, deletion of MIF promoted functional recovery (40). During SCI pathology, MIF promotes neuronal death by induction of oxidative stress and apoptosis, enhances immune cell infiltration into the spinal cord and stimulates pro-inflammatory cytokine production by microglia, leukocytes and astrocytes (38, 39, 41, 42). Here, plasma MIF levels were not different between HC, (s)aSCI and cSCI patients, which contrasts a study using chronic patients at >1 year post-SCI (43). This could be due to discrepancies in clinical characteristics and numbers of included SCI patients or the use of a different MIF detection platform. We hypothesize that MIF expression is elevated within the spinal cord lesion post-SCI, resulting in an inflammatory response and increased levels of CD74^+^ B cells that will lead to recruitment of CD74^+^ memory (IgG^+^) B cells into the injured spinal cord. In this way, inflammatory memory B cell responses are sustained and will aggravate the secondary injury phase. Future studies should focus on the exact role of CD74 and MIF in post-SCI B cell responses and investigate the possibility of therapeutically targeting these pathways.

There are some limitations to this study. A low number of SCI patients was included. This is due to the wide geographical spread of new SCI cases, challenges in the follow up of SCI patients and in the generation of PBMC collections, as PBMC isolation is not commonly performed in the SCI research field. Thus, our study provides an important first exploration of circulating immune responses and an interesting set of data that warrants further study in other patient cohorts. Further, absolute numbers of the included immune cell subsets should be analyzed. Additionally, the antigen specificity of the post-SCI memory and CD74^+^ B cell response remains unknown. Future research should focus on the potential autoreactivity of circulating B cells from SCI patients.

In conclusion, by performing high-dimensional immune profiling of SCI patients, this study provided more insight into the post-SCI systemic immune response. Overall, immune cells of SCI patients were shifted towards memory phenotypes, probably due to repeated immune activation, with most pronounced effects on B cells. Here, post-SCI B cell responses with elevated CD74 expression were observed, whereby IgG^+^ and IgM^+^ B cell levels correlated with injury severity. Thus, our results provide new potential B cell related targets for which the clinical, prognostic and therapeutic value for SCI management should be further studied.

## Data Availability Statement

The raw data supporting the conclusions of this article will be made available by the authors, without undue reservation.

## Ethics Statement

The studies involving human participants were reviewed and approved by Medical Ethics Committee of Hospital East Limburg (B371201317091), Academic Hospital Maastricht (METC13-4-079) and other institutional Medical Ethics Committees. The patients/participants provided their written informed consent to participate in this study.

## Author Contributions

JF, CL-G, BD, JD, DP and VS were involved in the study concept and design. JF, LB and EC acquired the data. JF and VS analysed and interpreted the data. All authors drafted and/or revised the manuscript for content. All authors read and approved the final manuscript.

## Funding

This work was supported by Hasselt University and a grant from the Wings for Life Spinal Cord Research Foundation (WFL-BE-23/17).

## Conflict of Interest

The authors declare that the research was conducted in the absence of any commercial or financial relationships that could be construed as a potential conflict of interest.

## Publisher’s Note

All claims expressed in this article are solely those of the authors and do not necessarily represent those of their affiliated organizations, or those of the publisher, the editors and the reviewers. Any product that may be evaluated in this article, or claim that may be made by its manufacturer, is not guaranteed or endorsed by the publisher.
