# Supplementary material for: Altered Circulating Immune Cell Distribution in Traumatic Spinal Cord Injury Patients in Relation to Clinical Parameters
Source: Front Immunol. 2022 Jun 28;13:873315. doi: 10.3389/fimmu.2022.873315 (PMC9273975; doi:10.3389/fimmu.2022.873315)
Supplement: Supplementary file 1 [file DataSheet_1.pdf]

Supplementary Material

Altered circulating immune cell distribution in traumatic spinal cord injury patients in relation to clinical parameters

Judith Fraussen, Lien Beckers, Charlotte C.M. van Laake-Geelen, Bart Depreitere, Jens Deckers, Erwin M.J. Cornips, Dieter Peuskens, Veerle Somers

Supplementary Table 1. Detailed information on SCI patients

Subject	Age	Gender	AIS score		Location injury	Aetiology	Surgery	Included time points
			t0	Later time points				
SCI-01	61	M	C	C at 3m	Cervical	Fall	Laminectomy	t3
SCI-02	56	M	C	C at 3w	Thoracic	Motor vehicle accident	Fixation (2x)	t3, t4
SCI-03	64	F	B	n.a.	Cervical	Fall	ACDF	t0
SCI-04	56	M	D	E at 1d	Cervical	Fall	Laminectomy	t0, t1, t2, t3, t4
SCI-05	64	M	A	A at 7m	Thoracic	Fall	Fixation	t0, t1
SCI-06	48	M	B	n.a.	Cervical	Motor vehicle accident	Halo-gravity traction, spondylodesis	t0, t1, t2
SCI-07	58	F	A	A at 4m	Thoracic	Fall	Fixation	t2
SCI-08	62	M	A	C at 12w	Cervical	Fall	Luxation, fusion, laminectomy	t0, t1, t2, t3
SCI-09	73	M	A	n.a.	Thoracic	Fall	Spondylodesis	t0
SCI-10	48	M	D	D at 3m	Cervical	Motor vehicle accident	Spondylodesis, ACDF	t2
SCI-11	58	M	A	A at 9m	Lumbar	Fall	Spondylodesis	t5
SCI-12	18	M	B	B at 12m	Cervical	Sports accident	Fixation, spondylodesis	t2, t3, t4, t5
SCI-13	67	M	D	D at 6m	Cervical	Fall	/	t1, t2, t4
SCI-14	61	M	D	D at 6m	Cervical	Fall	Laminectomy, spondylodesis	t3, t4, t5
SCI-15	68	M	D	E at 5d	Cervical	Sports accident	Fusion, laminectomy	t0, t1, t2, t3, t4
SCI-16	28	M	A	A at 4m	Thoracic	Gunshot	Laminectomy	t0, t1, t2, t3
SCI-17	54	M	C	D at 15m	Cervical	Sports accident	ACIF decompression	t0, t1, t3
SCI-18	60	M	D	E at 24m	Cervical	Fall	Fusion	t3, t4

Abbreviations: ACDF, anterior cervical discectomy and fusion; ACIF, anterior cervical interbody fusion; F, female; M, male; n.a. not available; d, days; m, months; w, weeks

Supplementary Table 2. Monoclonal antibodies used for flow cytometry

Marker	Fluorochrome	Clone	Company
CD19	BV421	HIB19	BioLegend
CD24	BV605	ML5	BioLegend
CD27	APC	M-T271	BioLegend
CD38	BV711	HIT2	BioLegend
CD74	PE	LN2	BioLegend
CD267	PE-Dazzle594	1A1	BioLegend
CD268	APC-Cy7	11C1	BioLegend
CD269	PerCP-Cy5.5	19F2	BioLegend
IgD	PE-Cy7	IA6-2	BioLegend
IgM	BV650	MHM-88	BioLegend
IgG	AF700	G18-145	BD Biosciences
IgA	FITC	IS11-8E10	Miltenyi Biotec
Fixable viability dye (FVD)	eFluor506	/	eBioscience

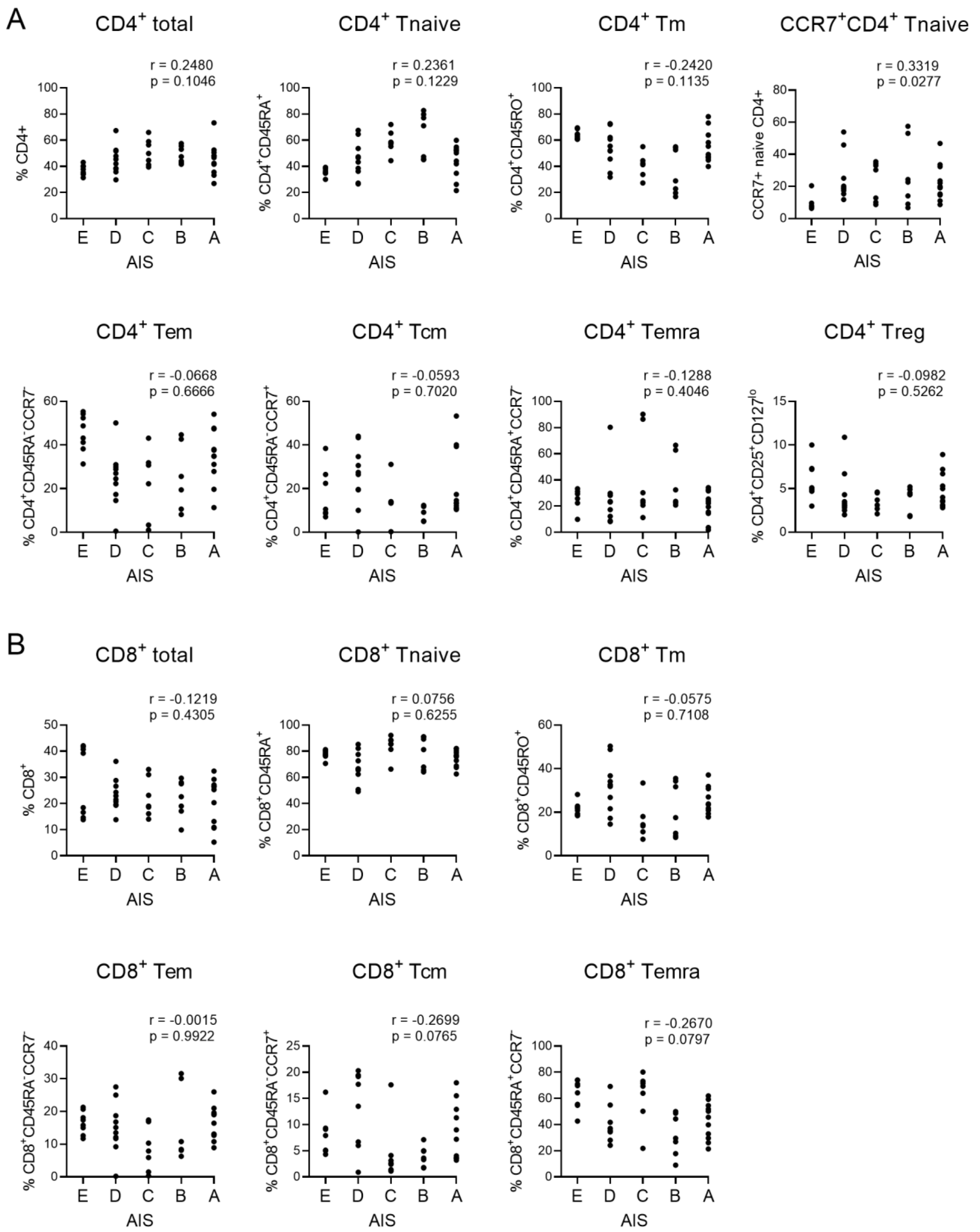

Supplementary Figure 1. Correlations of T cell subsets and disease severity. Correlation of the percentages of $CD4^+$ (A) and $CD8^+$ (B) total, Tnaive, Tm, Tem, Tcm, Temra and Treg with AIS score at sampling timepoint. r denotes the Spearman correlation coefficient (rho).

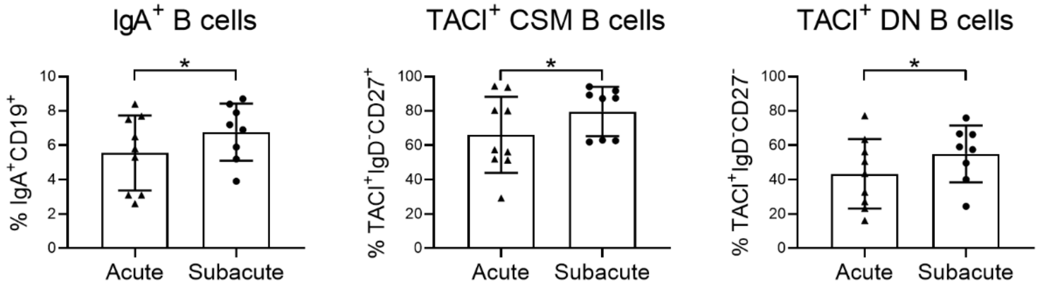

Supplementary Figure 2. Frequency of specific B cell subsets in the peripheral blood of acute and subacute SCI patients. Percentages of IgA^+ total B cells, $TACI^+$ CSM B cells and $TACI^+$ DN B cells are shown within the B cell population of acute SCI patients (n = 9) and subacute SCI patients (n = 8). Mean (bars) ± SD is depicted. * $p < 0.05$

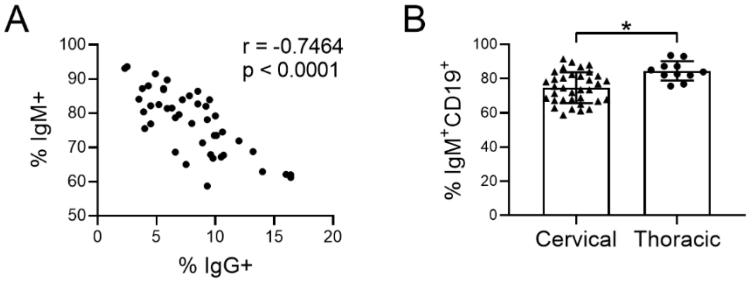

Supplementary Figure 3. IgM^+ B cells in correlation with IgG^+ B cells and in cervical and thoracic SCI patients. (A) Correlation of IgM^+ B cell percentages and IgG^+ B cell percentages. r denotes the Spearman correlation coefficient (rho). (B) Percentages of IgM^+ total B cells are shown within the B cell population of SCI patients with a cervical (n = 12) or thoracic (n = 6) injury, including 35 cervical and 11 thoracic samples collected at different (s)aSCI and cSCI time points. Mean (bars) ± SD is depicted. * $p < 0.05$
